# Advancing sexual and reproductive health outcomes in rural schools with the use of a sexuality education enhancement model: learners’ perspectives

**DOI:** 10.1016/j.heliyon.2022.e11189

**Published:** 2022-10-20

**Authors:** Ayobami Precious Adekola, Azwihangwisi Helen Mavhandu-Mudzusi

**Affiliations:** a Institute for Gender Studies, College of Human Sciences, University of South Africa, Pretoria, South Africa; b Department of Health Studies, College of Human Sciences, University of South Africa, Pretoria, South Africa

**Keywords:** Adolescents, Information-motivation-behavioural skills model, Learner, Rural-based schools, Sexual and reproductive health, Sexuality education, Systems theory

## Abstract

Despite evidence-based proof that sexuality education enhances school-going adolescents’ sexual and reproductive health, the high number of teenage pregnancies and sexually transmitted infections, including HIV, among young people in the rural areas of South Africa suggests that the school-based sexuality education programme in the rural areas might have less influence on learners due to various contextual factors in these settings. This phenomenological study used nine focus group interviews to explore the experiences of 84 school-going adolescents regarding sexuality education offered in their schools, located in the rural areas of the King Cetshwayo District, in Kwazulu-Natal, South Africa. The participants were purposively sampled and the transcribed data from the interviews were thematically analysed. The study revealed that the sexuality information provided to learners did not improve their motivation or encourage behaviour to act on the information provided. Additionally, the results highlighted environmental factors in the research setting that influenced the effectiveness of the sexuality education programme. To enhance its effectiveness, a sexuality education enhancement model was developed using elements of the systems theory and modified information-motivation-behavioural skills model. The model identifies the input resources needed to address issues of information, motivation and skills as well as the influencing environmental factors.

## 1 Introduction

Adolescents' path to healthy sexual and reproductive health (SRH) is a process that involves access to appropriate information on sexuality and a process to develop skills, attitudes and values regarding sexual consent, which can be addressed through sexuality education (Breuner and Mattson, 2016). Greenberg et al. (2017) argue that equipping school-going adolescents with knowledge of their sexuality will improve their sexual decision-making skills and empower them to clarify their values. The authors maintain that sexuality education deepens young people's understanding of the complex nature of human sexuality and fosters health-promoting attitudes. Likewise, Panchaud et al. (2019) note that the transition of adolescents to adulthood can be mediated by equipping them with SRH information and skills that will help them to have fulfilling relationships and attain SRH and well-being.

Several studies have shown that a well-implemented sexuality education is beneficial to learners. According to Keogh et al. (2018), sexuality education contributes to the development of positive attitudes and perspectives in young people towards SRH, relationships, gender issues, sexuality and human rights. On a related note, sexuality education fosters acceptance of other people's sexuality, equips learners with communication, negotiation and refusal skills, cultivates their critical thinking skills and improves their self-efficacy (Mavhandu et al., 2022; Baams et al., 2017; Ogolla and Ondia, 2019; Ponzetti, 2016).

Despite these benefits, several studies noted that the positive influence of sexuality education on young people was mostly realised in urban and semi-urban areas, while its impact is limited among school-going adolescents in rural areas (Odimegwu et al., 2018; Sommer and Mmari, 2015). Wanje et al. (2017) reckon that certain prevailing contextual factors in rural areas are responsible for the lesser influence of school-based sexuality education programmes on learners in rural areas. This was supported by Wood (2013), who indicates that factors such as cultural norms, beliefs, community actors and parental antecedents are likely to influence the effectiveness of the sexuality education programme in rural areas.

This prompted the researcher to conduct a study aimed to identify and describe the prevailing factors and determinants that could be hindering or enhancing the effectiveness of sexuality education at rural-based schools in King Cetshwayo District and what could be done to enhance the programme's effectiveness. The researcher conducted this study to explore the experiences of learners with regard to sexuality education being offered in their schools, because of the high rate of teenage pregnancy among school-going adolescents in the King Cetshwayo District, a rural area in KwaZulu-Natal, South Africa. The high prevalence of teenage pregnancy suggested that learners were engaging in unprotected sex, which also signifies that the sexuality education programme in these schools might not be effective. Guided by Scheerens’ (1990) method of theory generation, as well as Chinn and Kramer’s (2015) theory on the development process, as cited by McEwen and Wills (2019), the researcher developed a sexuality education enhancement (SEE) model, based on the research findings and the reviewed literature, using elements of the systems theory and a modification of the constructs of the information-motivation-behavioural skills model. The developed model aims to enhance the effectiveness of the school-based sexuality education programme in the research setting. The proposed model was refined and evaluated using the modified Delphi technique.

## 2 Information-motivation-behavioural (IMB) skills model

The IMB skills model is the theoretical framework that underpins this study. According to Fisher et al. (2014), the IMB skills model is generalisable to health behaviour programmes and has been applied successfully in different contexts, globally, to understand, predict and promote HIV prevention behaviours. This was supported by Bartholmae (2016), who argues that the IMB skills model can be used for understanding and promoting health-related behaviours. Likewise, the Sex Information and Education Council of Canada (SIECCAN) (2019) affirms that the IMB skills model is not only widely applicable to different population groups, but also well supported and suitable for effective sexual health-related studies and responsive intervention programmes. There are three basic constructs central to the IMB skills model, that is, information, motivation and behavioural skills (Rongkavilit et al., 2010).

Kiene et al. (2013) assert that an individual, who is well informed about HIV prevention, is well-motivated and has the necessary HIV prevention skills, will be capable to enact and maintain HIV preventative behaviours. In the context of sexuality education, SIECCAN (2019) argues that school-based sexuality education should provide relevant SRH information, motivational factors that promote sexual health behaviours and the behavioural skills needed to achieve sexual reproductive health and well-being. In line with this, Reis et al. (2011) posit that learners, who have acquired the relevant sexuality education information, motivation and skills, can change their attitude and, subsequently, their sexual behaviour. The IMB skills model implies that effective sexuality education should provide learners with relevant SRH-related information, foster their motivation to achieve SRH, and build their skills to initiate and maintain good SRH and well-being (Starks et al., 2017).

**Figure 1:**
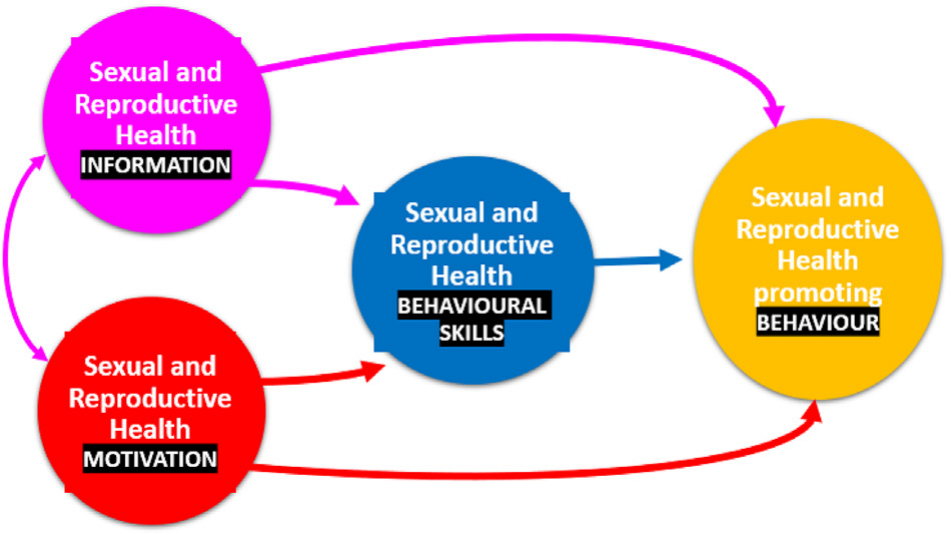
The information, motivation, behavioural skills model, adapted from Fisher et al. (2014).

Furthermore, the constructs of the IMB skills model could guide teachers in curriculum planning, delivery and evaluation (SIECCAN, 2019). In the same manner, Macapagal et al. (2016) indicate that the IMB skills model helps researchers to understand cognitive and behavioural antecedents of sexual risk among school-going adolescents. Several studies, however, proposed that the IMB skills model (shown in Figure 1) be modified to include an additional construct that addresses influencing environmental factors such as socioeconomic, cultural, and structural factors (Jiang et al., 2019; SIECCAN, 2019; Bartholmae, 2016). The authors reckon that a modified IMB skills model will expand the predictive ability of the model to include more variance in regard to adolescents’ health-promoting behaviours.

## 3 Systems theory

According to Yaşar (2017), education is a system made up of sub-systems, with interacting members that are open to environmental stress. This was supported by Oyebade (2010), who argues that issues related to education systems are best addressed using a systems approach. McEwen and Wills (2019) indicate that a system consists of different interacting, independent and organised elements, which function as a whole unit. In addition, the systems approach highlights the interactions of relevant components of a particular programme and how they support or undermine the achievement of its intended outcomes (Brent, 2019). This line of thought was supported by Gupta and Gupta (2013), who concur that using the systems theory approach in education could enhance stakeholders’ decision-making process about a particular teaching-learning situation. Therefore, these perspectives guided the researcher to adopt the systems theory in the development of the sexuality education enhancement (SEE) model.

**Figure 2:**
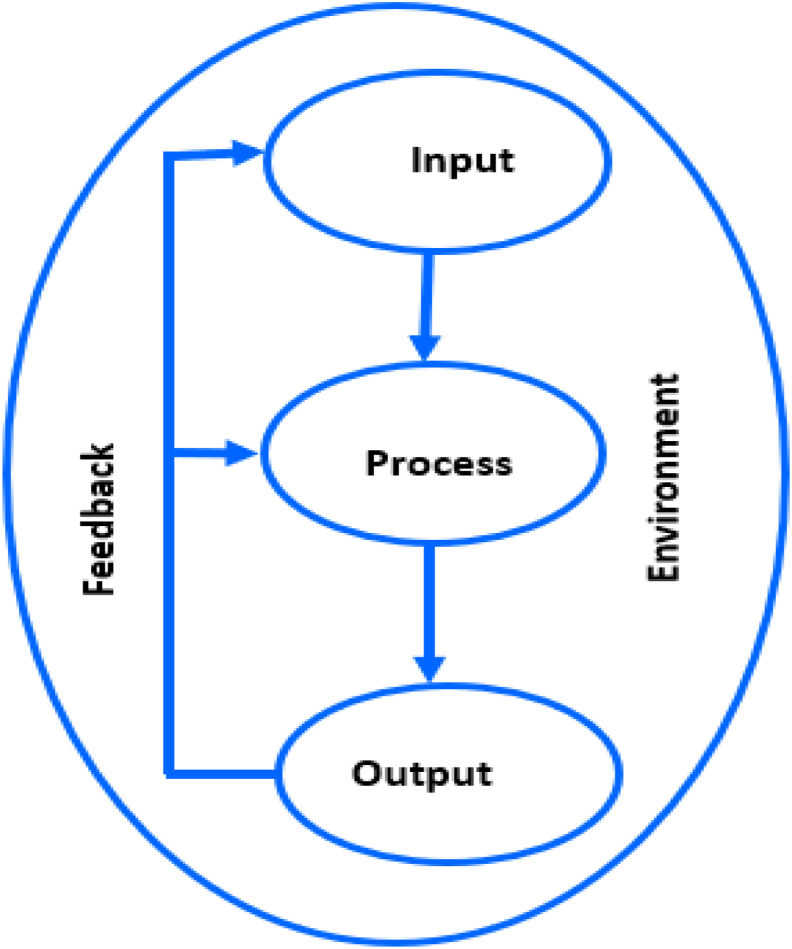
Systems theory adapted from Lunenburg and Ornstein (2012).

The elements of the systems theory (shown in Figure 2) include input, process, output and environment and these elements interact in a continuous feedback loop (Mania-Singer, 2017). For this study, the school-based sexuality education programme is a sub-system of the educational system, which is opened to the influence of prevailing environmental factors in the rural areas of the King Cetshwayo District.

## 4 Methodology

The methodology for this study was done in two phases, namely situational analysis and model development.

### 4.1 Situational analysis

#### 4.1.1 Design

This phenomenological study aimed to develop a model to enhance the sexuality education programme at schools in the rural areas of the King Cetshwayo District based on the analysis of school-going adolescents' lived experiences and perspectives regarding sexuality education offered at their schools. Polit and Beck (2017) explain that this study design allows a researcher to deeply understand the lived experiences shared by the participants and to make sense of the contexts in which the participants’ experiences took place. While the participants shared their personal experiences and perspectives regarding the sexuality education programme in their schools, the researcher listened and, thereafter, asked critical and clarifying questions to gain a better understanding of their experiences.

#### 4.1.2 Setting

This study was conducted in nine public secondary schools located in the rural areas of the King Cetshwayo District, in the KwaZulu-Natal province, South Africa. The sexuality education curriculum is offered as part of Life Orientation, which is a mandatory subject at all the selected schools.

#### 4.1.3 Sampling

The researcher used a non-probability purposive sampling technique on Grade 10 and 11 learners in the public schools situated in the rural areas of the King Cetshwayo District. This study was conducted in 2020 and learners, who met the pre-set inclusion criteria, formed the sample for this study. The inclusion criteria include being a Grade 10 or Grade 11 learner, aged between 14 and 19 years, residing in the rural areas of the King Cetshwayo District, fluent speaker of either or both English and isiZulu languages and willingness to be audio recorded. In addition, the learner should be ready to sign an informed consent form, coupled with obtaining parental consent, to participate in the study. For this study, 84 participants, made up of 49 female learners and 35 male learners, took part in the focus group interviews. This sample size was guided by data saturation, which, according to Saunders et al. (2018), happens when a researcher obtains first-hand data from participants that repeat the information that was previously obtained from other participants. This implies that a researcher cannot develop new ideas, themes and categories from the newly obtained data. Furthermore, data saturation occurs when the analysis of newly obtained data from participants does not yield relevant information to the research objectives (Mavhandu-Mudzusi, 2016). More than half of the participants (n = 49) are sexually active, with about sixty percent of the sexually active participants (n = 29) claiming that they had multiple sexual partners in the last three years. Most of the sexually active participants (n = 41) self-reported that they regularly used condoms, while the remaining eight sexually active participants claimed they used condoms sometimes. In addition, eight percent of the female participants (n = 4) reported that they had been pregnant before and a quarter of the participants (n = 21) reported they had no exposure to alcohol at the time of data collection.

#### 4.1.4 Data collection

The researcher used focus group interviews in the data collection process, which provided the researcher with rich information, because the participants freely narrated their experiences and perspectives. In addition, the focus group interviews provided the researcher with in-depth insights of sexuality education in the research setting. The focus group interviews took place at the selected schools, over five months, from March to July 2020. The researcher used a pilot study to refine the developed focus group interview guide that was used for mediation of the interview process. In line with Kvale’s (1996) interview guidelines, as cited in Qu and Dumay (2011), the researcher asked participants central questions such as: “From your experience as a learner in this school, what are your perceptions regarding sexuality education being offered at this school?” To obtain detailed and rich data from the shared experiences, the researcher further asked probing and prompting questions from the participants. The researcher audio recorded all the focus group interview sessions with the participants, with each session lasting between one-and-half to 2 hours. Furthermore, some of the focus group interview sessions took place after the COVID-19 lockdown regulations were relaxed, therefore, the researcher ensured that the Department of Basic Education's safety protocols – like social distancing, hand washing, sanitisation, wearing of facemask and the use of well-ventilated venues - were strictly adhered to during data collection process. The researcher used a high-quality audio recorder to capture the participants' shared experiences and perspectives during the focus group interview sessions to improve clarity and audibility.

Additionally, the researcher recorded his reflections and observations on the data collection process, as well as participants’ non-verbal cues, with field notes. The focus group interviews and the analysis of collected data were done iteratively until data saturation was reached.

#### 4.1.5 Data analysis

The researcher transcribed audio recorded data from nine focus group interviews, verbatim, into written text within two days. The nine transcripts obtained were kept in Microsoft Word format and were analysed using content analysis, as described by Bengtsson (2016). In addition, an expert independent coder analysed all the transcripts independently and the themes that emerged from his analysis were compared with that of the researcher, leading to a final table with three superordinate themes and several sub-themes.

#### 4.1.6 Trustworthiness

The researcher, guided by Lincoln and Guba (1985), used four criteria to ensure the trustworthiness of this study. These criteria are credibility, confirmability, dependability and transferability. In order to ensure that this study is credible, the researcher used member checking to verify that participants' experiences and views were accurately recorded. This was done on a continuous basis by allowing participants to listen to the recorded audio of the focus group interview sessions. Furthermore, the researcher gave some participants the transcripts of the interviews to confirm that the research findings are an accurate reflection of their experiences and perspectives. Besides ensuring credibility, the researcher enhanced confirmability and dependability of the study by using field notes to record the locations, dates and amount of time spent in the research setting. Additionally, audio recorded data of the group interviews were transcribed, verbatim, and independently verified by colleagues of the researcher. Likewise, an independent, expert coder analysed the transcripts to obtain independent themes. The researcher compared his emergent themes with the expert coder's independently determined themes to confirm accuracy and provided an audit trail of all the research activities that took place to ensure confirmability of the study. The researcher provided thick descriptions and rich details of participants' biographic details and the research context to ensure transferability of the study. In addition, the researcher provided detailed descriptions of the research process, setting, sample and robust details of the researcher's assumptions and experiences at every stage of the research process to further enhance transferability.

#### 4.1.7 Ethical considerations

The researcher sought and obtained ethical clearance from the University of South Africa and Provincial Department of Basic Education, in KwaZulu-Natal, to conduct this study. The researcher explained the aim, nature and the potential benefits of the study thoroughly to the participants. Furthermore, the researcher affirmed participants’ rights of refusal to participate in the research and emphasised to the participants that their participation in the study was voluntary and they may withdraw their participation at any time, without any negative consequence.

Before each focus group interview, the researcher obtained informed parental/guardian consent letters and assent forms from the participants, which were completed and signed by the parents/guardians and participants, respectively. To protect participants' identities, the researcher used pseudonyms in the transcripts and in the research reports. Furthermore, to enhance confidentiality, the researcher ensured the venues used for the group interviews were suitable, convenient and comfortable. While focus group interviews have peculiar challenges regarding anonymity, the researcher was guided by the approach of Sim and Waterfield (2019) to address this challenge. The researcher notified the participants about the public nature of group interviews and the challenges of ensuring anonymity during the group interviews. The researcher underscored the need for participants’ full cooperation to achieve the ethical requirement of anonymity. Before the interview started, the researcher gave the participants opportunity to withdraw their participation, if they were not comfortable regarding the anonymity issues. Furthermore, the researcher actively checked with the participants to confirm that they understood their responsibility regarding anonymity. The researcher also ensured that everyone participating in the focus group interviews, which were conducted during the COVID-19 pandemic, strictly adhered to all the safety protocols such as hand sanitisation, wearing of facemasks and keeping a distance of 2 m, and the researcher ensured that well-ventilated venues were used. Additionally, the researcher took measures to prevent unauthorised access to the audio-recorded and transcribed data obtained from the participants during the group interviews by keeping them safe in a secured electronic folder.

### 4.2 Model development

This phase of the research design focused on developing a model to enhance the delivery, implementation and effectiveness of the sexuality education programme in schools located in the rural areas of the King Cetshwayo District, KwaZulu-Natal. The development of the sexuality education enhancement (SEE) model was informed by the findings in the first phase of this study, which explored learners' experiences regarding sexuality education offered in their schools.

The conceptual framework that underpins this study is the IMB skills model, which guided the researcher in the development of the SEE model. Additionally, the findings from a literature review and elements of the systems theory were utilised to develop this model. The elements of systems theory used to formulate the SEE model include environment, input, process and output. The researcher followed Scheerens' (1990) method of theory generation, as well as Chinn and Kramer (2015)’s theory development process, as cited by McEwen and Wills (2019) and Rakhudu et al. (2017). This model development process includes concept analysis, synthesis, derivation and evaluation of the model. Furthermore, the researcher utilised a modified framework of the Delphi technique to refine and evaluate the model, as described by Hasson and Keeney (2011) and Crawford and Wright (2016).

#### 4.2.1 Concept analysis

Concept analysis aims to give meaning to a particular construct or concept by identifying all relevant definitions and characteristics (Polit and Beck, 2017). McEwen and Wills (2019) argue that the purpose of concept analysis is to recognise, clarify and define concepts that describe a phenomenon. The same authors maintain that concept selection is a critical step for conducting concept analysis. The researcher selected the sexuality education enhancement concept after in-depth literature review, because of its relevance to the phenomenon being studied. Therefore, as a point of departure, this study described and used the term, “sexuality education enhancement model”.

#### 4.2.2 Synthesis

Gray et al. (2017) refer to concept synthesis as the process where previously unrecognised concepts are named and described so that readers can understand the functions and meanings of the concepts. It refers to the development of new ideas and statements about a phenomenon or relationship through observation or by examining data to gain new insights (McEwen and Wills, 2019). Since synthesis starts with raw data, the researcher utilised the findings in the situational analysis phase of this study, as well as the literature review, IMB skills model and systems theory, to develop the SEE model. In addition, the constructs of the IMB skills model and elements of the systems theory were used to identify vital and relevant concepts and their inter-relationship in the formulation of the SEE model.

#### 4.2.3 Derivation

According to McEwen and Wills (2019), concept derivation guide researchers in the generation of new ways of thinking about the phenomenon of interest. This was supported by Gray et al. (2017), who assert that concept derivation could help researchers to identify concepts from other fields and, after establishing the concepts’ relevance, transpose them to a new field, in which it will be used. For this study, the researcher conducted concept derivation in both situational analysis and model development, as highlighted below:

The researcher described and interpreted participants' experiences and linked the study findings with reviewed literature. Additionally, the researcher adopted Walker and Avant's (2011) approaches, the IMB skills model and adopted elements of the systems theory in the development of the SEE model. To conclude the derivation process, the researcher utilised Chinn and Kramer’s (2015) modified criteria, as cited by McEwen and Wills (2019), in combination with a modified Delphi technique to evaluate and refine the model (Crawford and Wright, 2016; Hasson and Keeney, 2011).

#### 4.2.4 Model evaluation and refinements

The Delphi technique is a process used to measure a group of experts' judgments in order to make decisions, forecasts or assessing priorities (Gray et al., 2017). In this regard, Hasson and Keeney (2011) posit that the Delphi technique can be used to explore and predict attitudes of a group on a particular phenomenon. Anonymity, controlled feedback and aggregation of a group of experts' responses on a particular topic of interest are the basic characteristics of the Delphi technique (Keeney et al., 2011; Gray et al., 2017). Crawford and Wright (2016) and Keeney et al. (2011) propose that the panel of experts should be selected based on skills, knowledge and experience in the subject of interest. Therefore, the researcher purposively selected a group of five experts, based on their skills, knowledge and experiences on sexuality education, adolescents' SRH, the curriculum and model development. The researcher engaged the experts in three rounds of communication, before the model was finalised, based on the experts' feedback regarding the model's clarity, simplicity, generality, accessibility and importance. This process ensured that all unclear terminology and concepts in the model were clarified and conceptualised. Furthermore, the feedback obtained from the reviewers improved the model and its development. The panel of expert reviewers was satisfied that the overview and the elements of SEE model were well explained, with sufficient clarity.

In order to add rigour to the model evaluation process and further ensure trustworthiness, the researcher, guided by Chinn and Kramer’s (2015)’s modified criteria, shared the model with ten sexuality education teachers and five education experts for evaluation. The researcher utilised e-mail group and WhatsApp group chats to engage the reviewers for safety reasons due to the COVID-19 pandemic. This allowed the reviewers to share their perspectives and build consensual validation of the SEE model. The researcher compared the feedback from the teachers and education experts with the comments from the panel of expert reviewers. All the reviewers and evaluators felt that SEE model is user friendly, easily interpretable, relevant, adaptable to similar contexts and would positively enhance sexuality education programme in the research setting; thus, promoting adolescents' SRH and well-being.

## 5 Results

The findings of this study revealed that the barriers to effective school-based sexuality education programme in the research setting are learner-centred, educator-centred and community-centred. It also emerged from the study that certain factors and practices are likely to enhance sexuality education effectiveness in the schools located in the research setting. In addition, the study findings revealed interventions that may improve the effectiveness of sexuality education programme among learners in the rural areas the King Cetshwayo District of KwaZulu-Natal. The detailed findings of the study are discussed in reference to the structure of the SEE model below.

### 5.1 Description of sexuality education enhancement model

The three constructs of the IMB skills model align with the key learning objectives of sexuality education proposed by International Technical Guidance on Sexuality Education, which include knowledge, attitude and skills building (UNESCO, 2018). In addition, the IMB skills model was modified to include a new construct, namely environmental factors, based on this study's findings. The researcher developed the SEE model to achieve these (previously mentioned) objectives in the study setting. The SEE model entails provision of scientifically accurate, age-appropriate, contextually relevant and inclusive information to learners, to address attitude-related and motivational factors, and to cultivate the required and relevant behavioural skills to achieve sexual and reproductive health and well-being. Consequently, when school-going adolescents are well informed about SRH through sexuality education lessons, motivated to act and are equipped with relevant behavioural skills, they will be empowered to initiate, achieve and maintain SRH and well-being. The SEE model is inspired by the IMB skills model, because it addresses the psychological and environmental determinants of young people's behaviour, which can influence their attainment of SRH and well-being. Policymakers can use these constructs for planning, delivery and evaluation of the sexuality education programme in the study setting.

### 5.2 Purpose of sexuality education enhancement model

SIECCAN (2019) reckons that the goals of school-based sexuality education are to equip learners with the information, motivation and behavioural skills to enhance and attain sexual health and well-being. These goals are in line with the purpose of the SEE model. In this context, the overarching goal of the SEE model is to enhance the effectiveness of school-based sexuality education among learners in the rural areas of the King Cetshwayo District of KwaZulu-Natal. It aims to enhance educators’ pedagogical efficacy and empower learners to translate knowledge, acquired in sexuality education lessons, and their motivations into behavioural skills for initiating, achieving and maintaining SRH and well-being.

### 5.3 The structure of sexuality education enhancement model

The researcher utilised elements of the systems theory - made up of context, input, processes, output and outcome - in the development of the SEE model:

#### 5.3.1 Context

Context is the interrelated conditions in which a phenomenon exists or occurs (Bate, 2014) and it plays a critical role in intervention programming (Gray et al., 2017). Svanemyr, et al. (2015) argue that a useful sexuality education model should acknowledge the inter-level interactions and contexts of various determinants that influence adolescents' health outcomes. This guided the researcher to highlight three contextual levels for the SEE model, namely the global, national and district levels.

The SEE model is in line with the relevant Sustainable Development Goals (SDG 3, 4, 5, 16 and 17), which is aimed at achieving universal access to SRH services, HIV prevention, quality education, gender equality, good health and total well-being of young people (UNESCO, 2016). Furthermore, the model was guided by the global policy environment, for instance, UNESCO's International Technical Guidance on Sexuality Education (2018), the IPPF's Deliver + Enable Toolkit: Scaling-up Sexuality Education (2017), the IPPF Model for Sexuality Education (2010) and UNFPA Operational Guidance for Sexuality Education: A Focus on Human Rights and Gender (2014). Besides the global context, national policies such as the National Strategic Plan (2017–2022), the South African Department of Basic Education's Life Orientation Curriculum and Assessment Policy Statement (2011), and the Department of Basic Education National Policy on HIV, STIs and Tuberculosis (2017), guided the formulation of the SEE model. The implementation of the model should also be guided by these policies, because they provide a framework and direction for programming, delivery and implementation of school-based sexuality education in South Africa. In addition, the local context for the operationalisation of the model is the research setting, which includes all the schools located in the rural areas of the King Cetshwayo District, KwaZulu-Natal. The findings of this study at the schools located in the research setting form the basis for the model development.

#### 5.3.2 The input

Input relates to the human, financial, physical and information resources needed to carry out the transformational process in a system (Lunenburg, 2010). The findings of this study revealed that input resources such as training, multi-stakeholder collaboration, infrastructure and information resources are essential to enhance sexuality education in the research setting. While the provision of these input resources is necessary to create an enabling environment for school-based-sexuality education, they should target learners, teachers and the community actors. Such interventions should also address environmental factors that limit the access of young people to SRH services in the community.

##### 5.3.2.1 Training

The findings of the study showed the need for sexuality education teachers to undergo continuous professional training, which should be aimed at changing their attitudes and empowering them to be well grounded in the scientific facts that underpin sexuality education curricula as well as to improve their pedagogical competency. It was revealed from the lived experiences of the participants that some teachers were not comfortable and struggled with certain aspects of the sexuality education curriculum.

I do not think they are comfortable talking about some topics on sexuality. You can see it from the teachers' body language. They are not themselves because they don't go into details. They only teach us about disadvantages of sex like teenage pregnancy, diseases and so on. – (Anele, female, 17 years old)

It was further established that certain topics like contraception, sexual identities and sexual pleasure were skipped by the teachers, because they find them difficult to teach.

Sometimes they (teachers) are not at ease, with a little bit of awkwardness here and there when you ask questions. We don't demonstrate these things in the class probably they weren't comfortable with that, yeah like how to put a condom on and so on. – (Thabani, male, 17 years old)

LO teachers must not filter information during lessons. They must teach us everything as we get to the age of puberty. – (Zethembe, female, 17 years old)

This suggests that teachers should be continuously trained to acquire knowledge about the fundamental concepts of sexuality education curricula, ideology and pedagogy. In addition, it emerged from the analysis of research data that the Department of Basic Education needs to allocate more resources for training and workshops to build the capacity of sexuality education teachers in the study setting. This was supported by Vanwesenbeeck et al. (2015), who reported that teachers' motivation, attitude and skills to facilitate participatory and a learner-centred learning process are crucial to the effectiveness of sexuality education programmes. Therefore, the model proposes that the training provided to sexuality education teachers should equip them with sufficient information on sexuality education as prescribed by International Technical Guidance on Sexuality Education, adolescents' SRH, and strategies for effective teaching of sexuality education. Furthermore, educators' training should empower them to acquire information on sexuality education curriculum specifications, develop sexuality education lesson plans, deal with learners and parents’ discomfort, to enhance their personal confidence and composure in the classrooms, and ability to easily access resources on sexuality education.

This study further established that factors such as the teacher-learner age gap, religion, cultural background and health status, and educators’ experience can inhibit teachers’ motivation and commitment to deliver the sexuality education curriculum.

Some of our teachers are living with HIV, and some also suffer from guilty of what happened to them in their teenage years. Some of them have experienced teenage pregnancy and risky sexual practices so, when they are telling it to us, it is like they are blaming themselves. – (Mbalenhle, female, 17 years old)

They (the teachers) were not taught about their sexual life because the teachers of those days thought they were too young then but now they have to discuss everything with their learners which make them uncomfortable. So, I think they are not comfortable sometimes because they think they are exposing us to information that is not appropriate for us and that we may go and do ‘those things’. - (Nompilo, female, 18 years old)

The SEE model proposes training for educators that will boost their emotional, personal and social motivation regarding sexuality education programmes in their schools. These training programmes should address individual teacher's antecedents, perceptions, values and attitudes toward sexuality education, provide them with evidence-based information on the benefits of sexuality education, and address the identified personal biases, prejudices and discomfort in relation to the curriculum content of the sexuality education programme. This proposed SEE model is in line with the views of Bonjour and Van der Vlugt (2018), who note that well-trained teachers are an important factor in the delivery of quality and effective sexuality education.

##### 5.3.2.2 Multi-stakeholders’ collaboration

Besides the training of teachers, another significant finding that emerged from the study is the necessity to engage in multi-stakeholder collaboration in the study setting. It was noted from the analysis of research data that some parents and community actors, such as religious and cultural leaders, provided contradictory information and misinformation to school-going adolescents, which could undermine the key messages of the sexuality education curriculum.

“We all come from different homes, and our parents taught us differently. Even our teachers see things differently. In the community, there is a traditional practice, which encourages learners, and teenagers to get married while they are still in school, and they are allowed to fall pregnant. This practice does not agree with what we learnt in Life Orientation, but it happens here in this community”. – (Kwanda, male, 18 years old)

Some of the churches and parents do not accept what the teacher taught us in sexuality education. They think we should not be learning about sex at school, and

they do not approve of it because it does not agree with their religion. Some people in the community also think we are being encouraged to have sex, abortion, and “practice” gay. – (Mhlengi, male, 17 years old)

Apart from contradictory messages emanating from the community and learners' homes, the attitude of the teachers, community and parents, especially where there is disapproval of the sexuality education programme, can have a negative influence on learners’ motivation to engage in SRH-related behaviours and willingness to access SRH care and support services.

Some of our teachers are pastors too in their churches, and they think it is against their beliefs to discuss sexuality education with us. Some teachers think LO cannot change our behaviours no matter what they (teacher) do”. – (Nomonde, female 16 years old)

Certain people in the community believe that using protection during sex is bad because they want skin to skin but if a condom is not used you are at risk of getting diseases. Some cultural practices here don't believe in the usage of condoms when having sex. – (Phumulani, male 16 years old)

Furthermore, the study found that peer pressure and lack of access to non-judgemental SRH care and services are likely to demotivate young people from acting on the information received during sexuality education.

Sex is trending in the community among young people, and friends are pulling each other into doing it. Some do not follow the teachings in Life Orientation because of peer pressure. If all the people in your circle are smoking or having boyfriends, you will feel the odd one out, and it may look like something is wrong with you if you do not join them. – (Silungile, female, 17 years old)

The clinic is very far, and you cannot easily get free condoms too in the community. We are scared to speak about it to our parents or in the community because of the way you might be treated”. - (Mhlengi, male 17 years old)

This lack of social motivation may dissuade learners from initiating and maintaining SRH-related behaviours in the research setting. The study findings further indicated that socio-economic status, cultural norms and religious beliefs influence how learners receive school-based sexuality education and their attitude and motivation to act on the knowledge acquired during these sexuality education lessons.

The “blessers” are taking advantage of young boys and girls by buying them nice things and giving them money. Some parents even allow this to happen because they cannot provide for their children. *– (Sfanele*, female, 16 years old)

Additionally, the results indicate that some learners were inclined to rather follow their cultural beliefs when key messages that emanate from sexuality education do not align with cultural norms.

We end up not listening to our teachers because some believe in different cultures, and they are coming from different homes where they believe the things they are told by their parents. So what we do is to weigh things that we learnt from our teachers and things that we are taught by our family and come up with our conclusions”. – (Njabulo, male, 18 years old)

On a different note, this study revealed that parental support, rewards, as well as certain cultural beliefs, the activities of some religious organisations, provision of recreational facilities and access to friendly SRH services in the community could be enhancers of learners’ motivation to initiate and maintain positive SRH behaviours learnt in sexuality education.

Some cultural practices guard you to stay a virgin till you turn 21 years old or get married. It has a positive influence on sexuality education. My parents always say, get married first and then do those things. So, this helped me to be serious and wait for the right time and not to rush into things – (Nomonde, female, 16 years old)

In our community there are a lot of bad things that are happening. I have to focus at school and in sport because even sport teaches me many things. I have been learning about this sexual education with my team. I can say so”. – (Asande, female, 17 years old)

Therefore, the SEE model proposes that resources should be provided to collaborate on a continuous basis with key stakeholders such as Department of Basic Education district officials, parents, faith-based organisations, non-governmental organisations (NGOs), community leaders, learners and teachers to optimise the enhancers of sexuality education in the research setting and to address the stakeholder-related barriers to sexuality education. Likewise, the SEE model proposes that stakeholder engagement should stimulate local ownership of the sexuality education programme and promote socio-culturally acceptable ways of addressing stakeholders’ concerns. This study also highlights key areas of intervention in the research setting where input resources should be targeted and these inputs should include sexuality education campaigns, formation of help clubs, advocacy, eradication of poverty, and provision of information resources and infrastructure.

##### 5.3.2.3 Sexuality education campaigns

Resources should be made available to build community awareness about the impact of sexuality education on adolescents’ well-being and to address misconceptions about sexuality education in the communities.

We need to raise awareness to educate the community as well. This will help make the parents look at the things differently from the one they used to do. These may help sexuality education to be more effective in our school” – (Mandisa, female, 16 years old)

The awareness campaigns on sexuality education should extend to all the stakeholders in the community and could be initiated, managed and funded by the Department of Basic Education, in collaboration with NGOs and the private sector.

##### 5.3.2.4 Formation of help clubs

The schools and the local Department of Basic Education and NGOs should collaborate to form help clubs which are social groups in schools and in the community.

Yes, we need to form groups in the community. To teach other people in the community about sexuality education, HIV, and to stay away from unprotected sex. – (Kwanda, male, 18 years old)

This will likely enhance sexuality education among the learners and build social capital in the community. In addition, the help clubs will have defined goals and objectives to support school-based sexuality education programme. This may improve learners’ motivation and commitment to enact the skills learnt in the sexuality education programme; thus, enhancing their ability to attain SRH and well-being.

##### 5.3.2.5 Parent-teacher collaboration

The school management teams should implement a programme in the school academic calendar that will facilitate collaboration between parents and sexuality education teachers in the research setting.

The school must have meetings with the community and our parents so that they will allow us to practice what we learnt in sexuality education at school. I think Life Orientation teachers should be more specific on this one. They must organize meetings with parents to teach parents not to feel afraid to talk to their sons about this sexual intercourse. – (Sibonga, male, 16 years old)

This will empower parents to become active and constructive partners in school-based sexuality education. Furthermore, it will address concerns, misconceptions, misinformation and contradictory messages regarding sexuality education that emanate from learners’ homes.

##### 5.3.2.6 Advocacy

The use of respected professionals, social influencers and opinion leaders, with relevant experiences to reinforce important messages of sexuality education, can shape the opinions and social attitudes of young people and other stakeholders about the sexuality education programme. This may create an enabling and supportive environment that will assist the programme to achieve its outcomes in the community and could promote its acceptance. The SEE model proposes that the Department of Basic Education, district office and school managers should allocate resources toward sexuality education advocacy in their annual plan and budget.

##### 5.3.2.7 Eradication of poverty

The findings of the study indicate that addressing poverty in the research setting is critical to enhancing the effectiveness of the school-based sexuality education programme.

The government should help out with the people who live in poverty as this will reduce the number of people who are becoming prostitutes and getting pregnant” – (Mthokosizi, male, 17 years old)

The SEE model proposes that resources be allocated for the creation of community food bank and cash transfer programmes, which may potentially address household poverty, both in the short and long term. A collaborative effort between municipal authorities, NGOs, religious organisations, the private sector, the Department of Social Development, the Department of Basic Education and other relevant governmental agencies to address poverty could mitigate the negative impact that the socio-economic environment has on the effectiveness on the sexuality education programme.

##### 5.3.2.8 Infrastructure

While other studies have shown that availability of recreational and sporting facilities could enhance school-based sexuality education **(**Barkley et al., 2016), this study revealed that there is a lack of easily accessible sporting and recreational amenities available to young people in the research setting. This may likely impact the motivation of young people to act on the knowledge acquired in the sexuality education programme.

We need recreational facilities in our community that will keep us busy after school”. – (Nompilo, female, 18 years old)

The SEE model proposes the provision of accessible, youth-friendly, non-judgmental and non-discriminatory SRH facilities, and recreational and sporting infrastructure as the input resources that will enhance the outcomes of the sexuality education programme. The local government authority should invest in sport and recreational facilities to engage young people in skills-building activities after school hours; thus, strengthening the effectiveness of sexuality education programme in the research setting.

##### 5.3.2.9 Information resources

Apart from lack of supportive infrastructure, this study found that stakeholders’ access to information resources on sexuality education could be improved upon. The findings of this study indicate that, while the educators and parents desired positive SRH outcomes for learners, they lacked capacity to provide them with the necessary knowledge and skills on how to achieve those outcomes.

They tell you that getting pregnant is not right, but they will not tell you how to avoid getting pregnant- (Andiswa, female, 17 years old)

In addition to the scripted lessons and textbooks on sexuality education, the SEE model proposes that information and knowledge resources on sexuality education should be accessible to all stakeholders, using multiple sources like audio-visual resources, online portals, creative posters, informative pamphlets and other thematic activities that promote SRH and well-being of young people in the research setting. The local district offices of the Department of Basic Education can coordinate and provide these input resources.

Furthermore, the model also proposes utilisation of social media platforms to promote access to sexuality education information resources. Social media is interactive and could be attractive to young people for accessing SRH-related information resources and details of locations of SRH services in the community without compromising their privacy and confidentiality.

I think social media can also come in where maybe young people can be able to ask a sensitive and private question where confidentiality can be kept and all of that yes. – (Thando, male, 18 years old)

Additionally, social media platforms could be used for multi-stakeholder engagement initiatives and collaboration on the school-based sexuality education programme in the study setting.

## 6 Processes

**Figure 3:**
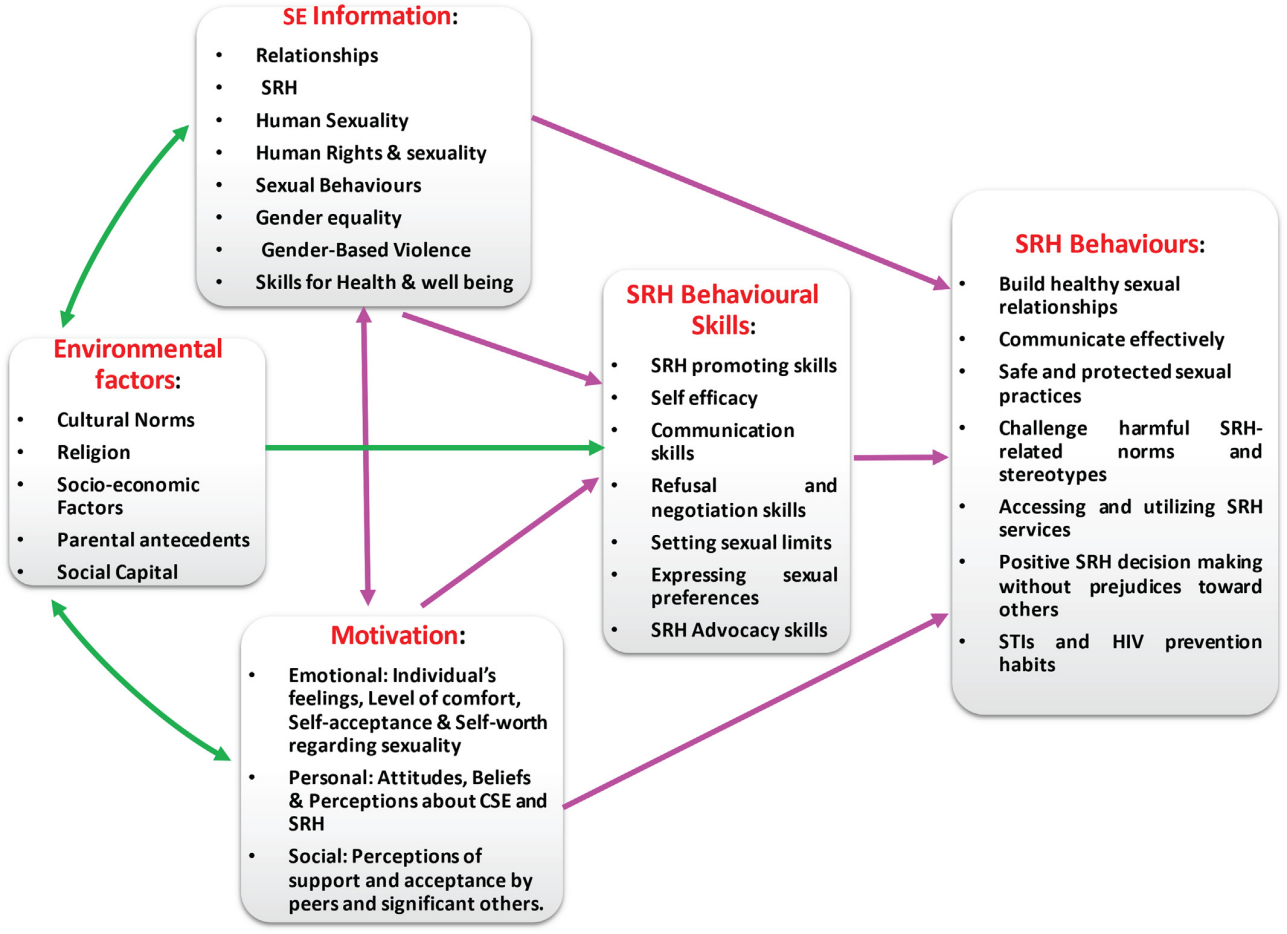
Processes for the sexuality education enhancement model.

The process refers to a mechanism that converts inputs into desired results. It consists of procedures or techniques that mediate the transition of inputs into outputs in systems theory (Scheerens, 1990). The SEE model used the constructs of the IMB skills model to develop its process unit. In addition to three essential constructs of the generalised IMB skills model, the SEE model's process is modified to have four constructs, based on the findings of this study (shown in Figure 3). This is significant, because the constructs of modified IMB skills model speaks to identified barriers and challenges to effective sexuality education in the study setting. Therefore, the following constructs, namely information, motivation, behavioural skills and environmental factors, are the mediating factors that underpin the process unit of the proposed SEE model which is aimed at transforming the input resources into the desired outputs.

The SEE model acknowledges, outlines and addresses key interrelated and interacting elements in the modified IMB skills model to enhance school-based sexuality education in the rural areas of the King Cetshwayo District. To access and utilise SRH care services and to develop complex skills for achieving sexual and reproductive health, young people need multiple information sources, and emotional, personal, and social motivation as well as the necessary objective and perceived skills. It emerged from the analysis of research data that there are learner-centred, educator-centred and community-centred factors that need to be addressed to enhance the school-based sexuality education programme in the study setting. Therefore, the overarching purpose of the SEE model's process unit is to use the input resources to address inhibiting environmental factors and to influence the information, motivation and behavioural skills of learners, educators and community actors positively regarding sexuality education. The SEE model predicts that the implementation of these processes will enhance the effectiveness of sexuality education in the schools in this research setting.

### 6.1 Information

The SEE model's information process calls for the provision of scientifically accurate, evidence-based, age-appropriate, contextually relevant information resources and materials that should be made available to educators, learners, parents and community members. This implies that there should be continuous training, community engagement and provision of information resources to inform stakeholders about the sexuality education curriculum.

### 6.2 Motivation

The findings of this study revealed multiple reasons why learners sometimes do not act on the information they receive in the sexuality education programme. The results indicated that motivation plays a critical role in whether a well-informed learner will be inclined to act on information in the classroom. It emerged from data analysis that lack of personal and emotional motivation could prevent learners from acting on information received in sexuality education.

I believe that it all start in the mind, the mindset of a person. If they've told themselves that okay I'm a teenager I ‘wanna’ experience things, there is nothing you can do about that, that's my opinion. - (Velisiwe, female, 18 years old)

Furthermore, this study established that providing learners with sexuality education information is not sufficient to achieve positive SRH behavioural changes; learners also have to be motivated. In addition, the results indicated that perceived lack of social support from both educators and peers, and the psychological status of learners have an impact on their motivation.

To keep the stress out of ourselves we do things we should not do normally so that we can forget about our stress. When you are stressed, you may not even remember what you learnt in the class, you just want to feel good and that why we do such things like smoking, drugs and sex to forget. – (Mthokosizi, male, 17 years old)

“Many teenagers do it just to keep their friendship even thou they know that what they are doing is bad”. – (Sisanda, female, 17 years old)

It was further noted from the study findings that providing learners with sport and recreational facilities, integrating certain cultural beliefs and accentuating the rewards of adopting positive choices could enhance their motivation to initiate and maintain positive SRH behaviours learnt in schools. Besides learners’ lack of motivation, the study findings revealed that a percieved lack of motivation on the part of some sexuality education teachers has prevented them from teaching the sexuality education curriculum in a comprehensive and professional manner, because the messages conflict with their personal antecedents and beliefs.

Most of our teachers like to emphasize abstinence from sex than telling us how to protect ourselves. They judge very much even if you date a guy they judge you that you are so young. In some lessons, the teacher just wants to do all the talking and teach us what to do to be safe. – (Snegugu, female, 17 years old)

Consequently, the SEE model's motivation process calls for sexuality education curriculum planners and teachers to focus on the motivation of learners to act on the sexuality education information received in the classroom that would improve their SRH. Similarly, the training and capacity-building resources for educators should target and address their motivation and attitude. Furthermore, the SEE process requires provision of infrastructure like recreational sports amenities and accessible, youth-friendly and non-judgemental SRH services to enhance young peoples' motivation to initiate and maintain positive SRH behaviours. The model's motivation process predicts that a well-informed and motivated adolescent is likely to act on the sexuality education knowledge received in the classroom.

### 6.3 Behavioural skills

The findings of this study revealed that there is a gap between the information provided to learners and the behavioural skills possessed by learners to act on the acquired information.

The results of the data analysis further indicated that sexuality education lessons, in the research setting, focused more on sharing information, with little or no emphasis on cultivating the relevant and necessary behavioural skills.

As much as they are teaching us about these sexual life, we still become pregnant as teenagers. – (Veliswe, female, 18 years old)

Therefore, the SEE model’s behavioural skills process advocates that all input resources should be harnessed to enhance educators and learners’ self-efficacy and objective abilities. The model demands that every input resource should focus on building the sexuality education-related skills of learners, educators and parents. Additionally, teachers' training should be designed to improve teaching and learning strategies around behavioural skills development. The SEE model argues that a well-informed, well-motivated educator requires the necessary pedagogical and behavioural skills to facilitate sexuality education teaching effectively and to achieve the desired behavioural outcomes in adolescents.

### 6.4 Environmental factors

The results indicated that there are environmental factors like poverty, cultural and religious norms, parental antecedents and community attitudes that influence the causal pathways of the constructs of the IMB skills model, which underpins the SEE model's processes in the study setting. Therefore, the environmental construct of the SEE processes seeks to create an enabling environment in order for the school-based sexuality education programme to be effective. The socio-economic status of learners may influence the motivation and behavioural skills constructs of the SEE model. The analysis of the research data revealed that learners, who came from poor homes, are more likely not to be motivated to act on knowledge received in classroom during sexuality education lessons, because they engage in transactional sexual activities for money and material gain.

Within the community, life is difficult for young people, and there is a temptation to do things that will make you comfortable. Schoolgirls want to have sugar daddies from mining company, and this is common in our community. These behaviours may not agree with what we learnt in Life Orientation, but life is not fair. You have to survive and look after yourself. – (Lethu, male, 17 years old)

Furthermore, the results from this study indicated that other social issues, such as substance abuse, have an influence on learners’ motivation and ability to act on sexuality education knowledge obtained in school. Learners, who engage in substance use, are likely to act contrary to the SRH information received in sexuality education.

We have a problem with drugs, and learners have access to these drugs in the community. Alcohol abuse is common in the community –Nomcebo, female, 17 years old)

In addition, the study findings revealed that the quality of information provided to learners and the teachers' motivation to adhere to the curriculum contents are negatively impacted by their cultural background. Similarly, the research findings showed that the cultural antecedents of the learners influenced how they respond to the SRH knowledge acquired in school. The results also revealed that some teachers skipped key parts of the sexuality education curriculum, because it conflicted with their cultural beliefs. The study established that certain cultural practices in the community are likely to negatively impact learners’ SRH and well-being, regardless of the sexuality education knowledge provided in the classrooms.

In addition, the study revealed that religious beliefs might have both a negative and a positive influence on learners regarding sexuality education. The study established that religious beliefs are likely to shape the views of parents, teachers, learners and the community, as a whole, towards the school-based sexuality education programme.

The SEE model processes highlight the need to address environmental factors that influence sexuality education in the research setting. The model further asserts that the provision and processing of the allocated input resources, to address these environmental factors, will enhance sexuality education in the rural areas of the King Cetshwayo District.

## 7 The output

Van den Bekerom et al. (2017) see output as the end product that follows systematic processing of input. The researcher anticipates that identified input resources, identified in the SEE model, will be considered and adequately allocated. In addition, it is expected that the modified IMB-based processes of the SEE model will be followed, as guided by International Technical Guidance on Sexuality Education, the National Strategic Plan and the South African Department of Basic Education policies. The expected output of the SEE model is effective delivery and implementation of the sexuality education programme in the study setting. This may lead to improved SRH behaviours such as delayed onset of sexual activity, reduced incidence of HIV and STI infections, increased access and utilisation of SRH services, reduced incidences of teenage pregnancy, reduced HIV and gender-related stigma and discrimination as well as acceptance of diverse gender norms.

### 7.1 Feedback

The SEE model's feedback provides for a monitoring and evaluation process, whereby the model gathers information about its performance. According to Oyebade (2010), feedback processes allow a particular system to self-regulate and self-direct. This is supported by SIECCAN (2019), which emphasises that monitoring and evaluation of the sexuality education programme is needed to ensure that its implementation is consistent with the fundamental principles of the programme and to determine if it is achieving its stated objectives. The researcher calls for the design of a uniform tool to obtain standard feedback from learners, teachers, parents and the school managers. This will provide the information needed by the Department of Basic Education's policymakers, administrators, school management teams and educators to understand the impact of the model and to use the data to improve the sexuality education programme in the study setting. The feedback data should be collected, at the school, from teachers, learners and, at the community level, from parents and other community actors. The SEE model proposes that feedback at the school level should take place regularly. The collected data could guide teachers in identifying gaps between the four constructs of the SEE model processes in relation to the implementation of the curriculum. Furthermore, it could help them to prepare a responsive intervention to address the observed gaps.

The objectives of the feedback's tools are to ascertain:

• The accuracy and relevance of the sexuality education information provided to the learners.
• If the contents of the curriculum and its implementation are comprehensive, inclusive, human rights-based, gender-sensitive and address gender-based violence.
• If it focuses on providing adolescents with SRH information, building motivation and developing the SRH behavioural skills of learners.
• If the programme creates awareness and engagement about influencing environmental factors that could impact sexuality education outcomes.
• If sexuality education lessons are mediated by well-informed and well-motivated teachers, who are equipped with the necessary pedagogical and behavioural skills.
• If curriculum implementation is guided by a balanced approach that promotes positive SRH habits and prevention of negative SRH outcomes.

## 8 Summary of the sexuality education enhancement model

**Figure 4:**
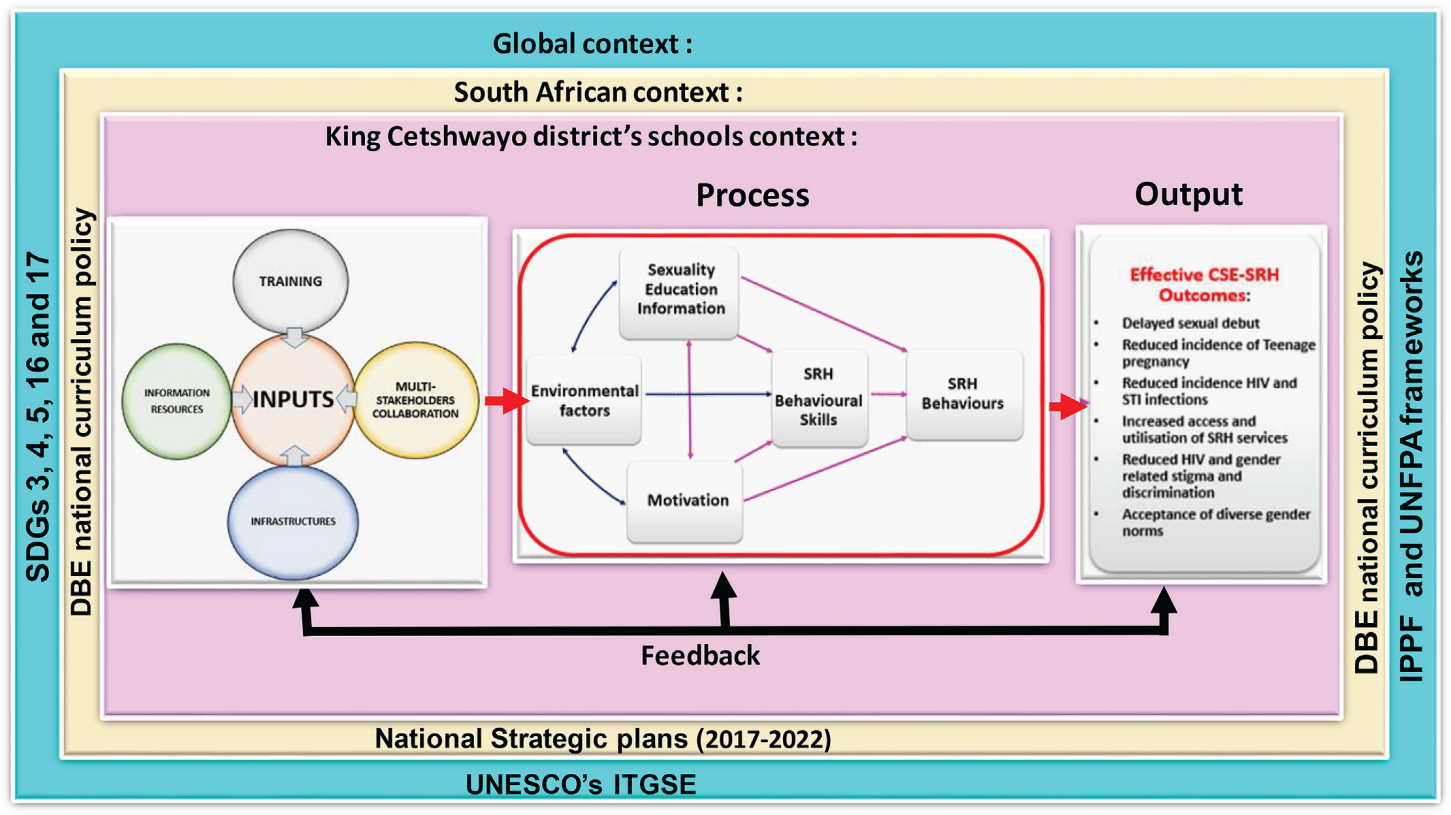
The sexuality education enhancement model (SEE model).

The SEE model consists of the following elements: a multi-level context (global, South African and King Cetshwayo District level); input (information resources, training, infrastructure, and multi-stakeholder collaboration); processes (information, motivation, behavioural skills, and environmental factors); and output (enhanced sexuality education programme, improved access and uptake of SRH services, improved SRH behaviours, improved adolescents’ SRH outcomes) as shown in Figure 4. The connecting arrows represent the inter-relationships between different components of the model. The IMB skills-based transformative processes of the model will utilise the input resources to produce enhanced sexuality education outcomes, which may lead to improved adolescent SRH and well-being in the study setting. The feedback component of SEE model gathers information on how well the model functions and how to modify it for enhanced effectiveness. All the elements of the SEE model need to function properly to achieve enhanced and effective sexuality education programme in the research setting.

## 9 Assumptions of sexuality education enhancement model

The SEE model assumes that, if the necessary resources are allocated for the sexuality education programme in the study setting - to address issues relating to information, motivation, behaviour and the environment - then the quality of school-based sexuality education programme being offered will be enhanced, leading to improved overall SRH and well-being of school-going adolescents in the research setting. In addition, the SEE model assumes that the quality of the sexuality education information provided to learners, the processing of this information by learners, the motivation of learners and teachers, and the behaviour of learners are affected by the prevailing environmental contexts in the research setting. Furthermore, the model assumes that a well-informed, well-motivated learner, who possesses all the necessary behavioural skills provided by sexuality education would be more inclined to initiate and maintain positive SRH behaviours leading to positive SRH outcomes. Conversely, learners, who lack the relevant SRH information, motivation and the necessary behavioural skills, are unlikely to engage in positive SRH behaviours.

## 10 Implications of the sexuality education enhancement model

The SEE model implies that the school-based sexuality education programme in the study setting will be effective when identified input resources are made available and are utilised to provide accurate sexuality education information, build motivation, and cultivate the skills of learners to initiate and maintain positive SRH behaviours and outcomes. Furthermore, The SEE model calls for the sexuality education curriculum to be mediated and facilitated by well-informed, motivated and skilled educators.

Another implication of the SEE model is that it calls on relevant stakeholders, such as policymakers in the Department of Basic Education, to recognise and address environmental factors that affect the effectiveness of school-based sexuality education in the research setting. This can be done through continuous professional development of the educators responsible for delivering the sexuality education programme. Therefore, the model implies that, when both teachers and learners are well informed, motivated and equipped with the necessary skills, and the interfering contextual factors were addressed, the sexuality education programme in the research setting will be effective and achieve its desired outcomes.

In addition, the SEE model implies that the ultimate objective of the school-based sexuality education is to empower learners to achieve positive SRH outcomes and well-being. The SEE model calls for a paradigm shift among all the stakeholders, such as the Department of Basic Education policymakers, curriculum planners and educators, as well as parents, learners and other community actors, regarding school-based sexuality education in the research setting.

## 11 Limitation of the study

For this study, the researcher adopted a non-probability and purposive sampling technique in the selection of the participants. Therefore, learners, whose experiences and perspectives might be different from the study participants’ experiences, could have been left out of the study. In addition, this study left out the experiences and views of parents and educators, but explored only the lived experiences and perspectives of learners in the study setting. The data collection was conducted by the researcher who is an adult male, it is possible that the perceived gender of the interviewer to have impact on the honest response to the interview questions. Furthermore, part of the data collection process for this study took place during the COVID-19 pandemic; therefore, the anxiety among the learners, who participated in the study, might have affected how they shared their experiences and views. On this basis, the interpretation of these research findings should take into account these limitations.

## 12 Conclusion

The SEE model was developed from the findings of this study. The model development was guided and supported by the IMB skills model, using elements of the systems theory. The model supports all the critical factors regarding school-based sexuality education addressed at global, national and district levels. The basic components of the SEE model are input, a modified IMB skills-based process, output and feedback. The model upholds that scientifically accurate information, motivation and behavioural skills influence learners' abilities to initiate and maintain SRH and well-being. It advocates for the provision of input resources to address environmental factors that influence information, motivation, and the behaviour of school-going adolescents and teachers to enhance the quality and effectiveness of the sexuality education programme in the King Cetshwayo District, South Africa. The SEE model's ultimate goal is to empower learners to initiate, engage and maintain positive SRH behaviours and outcomes through an effective school-based sexuality education programme in the research setting.

## Declarations

### Author contribution statement

Ayobami Precious Adekola, Ph.D: Conceptualised and designed the study; collected the data; Analyzed and interpreted the data; Wrote the paper.

Azwihangwisi Helen Mavhandu-Mudzusi, Ph.D: Supervised the study, analysed and interpreted the data

### Funding statement

This research did not receive any specific grant from funding agencies in the public, commercial, or not-for-profit sectors.

### Data availability statement

Data included in article/supp. material/referenced in article.

### Competing interest statement

The authors declare no conflict of interest.

### Additional information

No additional information is available for this paper.
